# Maternal Mediterranean Diet Adherence During Pregnancy and Autism-Related Traits in Preadolescence: A Sex-Stratified Analysis

**DOI:** 10.3390/nu18142256

**Published:** 2026-07-10

**Authors:** Spyridon N. Karras, Maria Dalamaga, Maria Kypraiou, Vikentia Harizopoulou, Antonios Vlastos, Marios Anemoulis, Neoklis Georgopoulos, Georgios Mastorakos, Dimitrios G. Goulis

**Affiliations:** 1 Laboratory of Biological Chemistry, Medical School, Aristotle University, 55535 Thessaloniki, Greece; 2 Department of Biological Chemistry, School of Medicine, National and Kapodistrian University of Athens, 10679 Athens, Greece; madalamaga@med.uoa.gr; 3 Assisting Nature Centre of Reproduction and Genetics, 57001 Thessaloniki, Greece; 4 Department of Midwifery, Faculty of Health and Caring Sciences, University of West Attica, 12243 Athens, Greece; vikentiaharizopoulou@hotmail.com; 5 Medical School, Aristotle University, 55535 Thessaloniki, Greecemariosanemoulis@hotmail.com (M.A.); 6 Division of Endocrinology, Department of Internal Medicine, School of Health Sciences, University of Patras, 26504 Patras, Greece; 7 Second Department of Surgery, Medical School, Aretaieio Athens Hospital, National and Kapodistrian University of Athens, 11528 Athens, Greece; 8 Unit of Reproductive Endocrinology, 1st Department of Obstetrics and Gynecology, Medical School, Faculty of Health Sciences, Aristotle University of Thessaloniki, 54124 Thessaloniki, Greece; dgg@auth.gr

**Keywords:** Mediterranean diet, pregnancy, autism-related traits, communication, neurodevelopment, birth cohort, adolescence, sex differences

## Abstract

**Background:** Maternal nutrition during pregnancy is increasingly recognized as an important determinant of offspring neurodevelopment. The Mediterranean diet has been associated with favorable cognitive and behavioral outcomes in children; however, evidence regarding autism-related traits remains limited, particularly during late childhood and adolescence. **Objective:** To investigate the association between maternal adherence to the Mediterranean diet during pregnancy and autism-related traits in preadolescent offspring in the KLOTHO birth cohort. **Methods:** This exploratory analysis included 96 adolescents (49 boys and 47 girls) from the KLOTHO birth cohort. Maternal adherence to the Mediterranean diet during pregnancy was assessed using a Mediterranean Diet Score derived from a food frequency questionnaire administered during gestation. Autism-related traits were evaluated during preadolescence using a parent-completed questionnaire that assessed social and emotional abilities, communication skills, cognitive characteristics, special interests, and motor skills. Associations were examined using Spearman’s correlation analyses and sex-stratified linear regression models adjusted for offspring body mass index, sleep duration, and physical activity, all assessed during the preadolescent follow-up examination. **Results:** Boys exhibited higher communication (3.92 ± 3.90 vs. 1.70 ± 2.89, *p* = 0.026), cognitive (5.76 ± 3.80 vs. 3.33 ± 2.99, *p* = 0.014), special-interest (2.64 ± 3.29 vs. 1.04 ± 2.16, *p* = 0.046), and overall autism-related trait scores (19.08 ± 13.92 vs. 10.15 ± 8.57, *p* = 0.009) compared with girls. In the overall cohort, maternal Mediterranean Diet Score was not associated with autism-related traits. Among boys, higher maternal Mediterranean Diet Scores were associated with lower communication-related trait scores (ρ = −0.442, *p* = 0.027). In multivariable analyses, the inverse association remained consistent in direction but did not retain statistical significance after adjustment for body mass index, sleep duration, and physical activity. Sensitivity analyses incorporating offspring KIDMED scores yielded similar findings. **Conclusions:** Higher maternal adherence to the Mediterranean diet during pregnancy was associated with lower communication-related trait scores in male offspring. These findings should be considered exploratory and require confirmation in larger prospective cohorts.

## 1. Introduction

Autism spectrum disorder (ASD) is a complex neurodevelopmental condition characterized by persistent difficulties in social communication and social interaction, accompanied by restricted, repetitive patterns of behavior, interests, or activities [1,2]. Recent evidence suggests that autism-related characteristics are not restricted to clinically diagnosed individuals but are continuously distributed throughout the general population [3,4]. Consequently, autism-related traits can be evaluated dimensionally, providing valuable insights into neurodevelopmental variation even among otherwise healthy children and adolescents [1,3]. The prevalence of ASD has increased substantially during recent decades, with current estimates suggesting that approximately 1–2% of children worldwide meet diagnostic criteria. Although improved awareness and diagnostic practices contribute to this increase, growing attention has focused on environmental and prenatal factors that may influence neurodevelopmental trajectories.

The etiology of autism-related traits is considered multifactorial, involving complex interactions between genetic susceptibility and environmental exposures occurring during critical developmental periods [2]. Among environmental influences, the intrauterine environment is increasingly recognized as a key determinant of long-term neurodevelopment [1,2,3,4,5]. Pregnancy represents a period of rapid fetal brain growth characterized by neuronal proliferation, migration, synaptogenesis, and myelination. Maternal nutritional status plays a fundamental role in supporting these processes by providing substrates and bioactive compounds required for normal brain development [6,7,8,9]. Consequently, maternal dietary patterns during pregnancy have emerged as potentially modifiable determinants of offspring neurodevelopmental outcomes [10,11].

Particular attention has been directed toward the Mediterranean diet, one of the most extensively studied dietary patterns worldwide [12,13]. The Mediterranean diet is characterized by high consumption of fruits, vegetables, legumes, whole grains, nuts, olive oil, and fish, moderate intake of dairy products, and relatively low consumption of red and processed meats. This dietary pattern provides a rich source of nutrients and bioactive compounds known to support neurological development, including folate, omega-3 polyunsaturated fatty acids, antioxidants, vitamins, minerals, and polyphenols [14,15].

Several biological mechanisms support a potential association between maternal Mediterranean diet adherence and offspring neurodevelopment [16,17]. Beyond providing adequate amounts of folate and long-chain omega-3 polyunsaturated fatty acids, this dietary pattern is characterized by a high intake of fruits, vegetables, legumes, nuts, olive oil and whole grains, resulting in increased consumption of antioxidants and (poly)phenolic compounds while reducing exposure to ultra-processed foods and pro-inflammatory dietary components. Recent prospective cohort studies suggest that higher maternal adherence to the Mediterranean diet is associated with more favourable cognitive, behavioural and neurodevelopmental outcomes in offspring, although evidence regarding autism-related traits remains limited and inconsistent. Proposed mechanisms include modulation of maternal systemic inflammation, oxidative stress, placental function, epigenetic programming and fetal neuronal maturation.

Folate is essential for DNA synthesis, methylation processes, and neural tube development, whereas omega-3 fatty acids, particularly docosahexaenoic acid (DHA), contribute to neuronal membrane integrity and synaptic function [18]. Furthermore, the anti-inflammatory and antioxidant properties of the Mediterranean dietary pattern may reduce maternal oxidative stress and inflammatory responses during pregnancy, both of which have been implicated in altered fetal brain development [16,17,18]. Accumulating data indicate that dietary exposures during pregnancy may shape fetal brain development via epigenetic pathways influencing gene expression and neural patterning [17]. Several cohort investigations have documented links between healthier maternal eating patterns and more favorable cognitive, behavioral, and emotional trajectories in children. Specifically, greater maternal adherence to a Mediterranean-style diet in pregnancy has been associated with enhanced cognitive outcomes, stronger executive function, fewer behavioral problems, and better psychosocial development throughout childhood [5,6,7,8,9]. However, evidence specifically examining autism-related outcomes remains limited and inconsistent [19,20,21].

The KLOTHO birth cohort offers a unique opportunity to investigate these questions within a prospective framework. Maternal dietary information was collected during pregnancy, and offspring were subsequently evaluated approximately 12 years later, allowing assessment of long-term neurodevelopmental outcomes. In addition, detailed information regarding lifestyle characteristics and dietary habits during adolescence permits consideration of potential confounding factors.

Therefore, the present study aimed to investigate the association between maternal adherence to the Mediterranean diet during pregnancy and autism-related traits in preadolescent offspring participating in the KLOTHO birth cohort. We additionally examined whether these associations differed according to offspring sex, given known sex differences in autism-related characteristics and neurodevelopmental vulnerability [1,2,3,4]. We hypothesized that higher maternal adherence to the Mediterranean diet during pregnancy would be associated with lower autism-related trait scores in offspring, particularly among boys.

## 2. Methods

### 2.1. Study Design and Participants

This work forms part of the KLOTHO cohort, a prospective, observational birth cohort based in Thessaloniki, Greece, designed to examine how early-life exposures shape long-term health outcomes in children. The present study represents a secondary exploratory analysis of an ongoing prospective birth cohort. Consequently, the sample size was determined by the availability of complete exposure and outcome data rather than by an a priori statistical power calculation.

Participants were pregnant women initially enrolled at the Maternity Unit of the First Department of Obstetrics and Gynecology, Aristotle University of Thessaloniki, Greece, from January through December 2011. Eligible pregnancies were singleton and reached term delivery (37–42 weeks). Women with major chronic illnesses or pregnancy complications known to influence fetal development were excluded. Following data collection during pregnancy, offspring were reassessed in early adolescence; those with complete dietary-pattern and psychological-assessment data were retained for analysis (Supplementary Figure S1), while participants missing key exposure or outcome data were excluded. Ethical approval was granted by the Bioethics Committee of Aristotle University of Thessaloniki (no. 1, 19 December 2011), with the study conducted in line with the Declaration of Helsinki. All participants, along with parents or legal guardians of minors, provided written informed consent.

### 2.2. Maternal and Adolescent Assessments

Maternal characteristics recorded included age at delivery, pre-pregnancy BMI, smoking status, and educational attainment, the latter serving as a proxy for socioeconomic status; because broader socioeconomic data were incomplete across the cohort, they were excluded from the final adjusted models. During follow-up, adolescents underwent anthropometric evaluation via standardized methods: height and weight were recorded to derive BMI (kg/m^2^), alongside waist, thigh, and head circumference measurements, all obtained by trained staff following consistent protocols. Lifestyle factors were captured through a structured questionnaire, including average daily sleep duration, while habitual physical activity was quantified using the Physical Activity Questionnaire (PAQ) composite score [21].

Maternal Mediterranean diet adherence was assessed prospectively during pregnancy and represented the primary exposure variable. In contrast, offspring BMI, sleep duration, and physical activity were measured during the adolescent follow-up approximately 12 years after birth and were considered contemporaneous covariates. Maternal age, pre-pregnancy BMI, smoking status, and educational level were initially evaluated as potential confounders. Because maternal educational data were incomplete and the number of observations available for multivariable analyses was limited, inclusion of additional maternal variables substantially reduced statistical power and increased the likelihood of model overfitting. Therefore, the final adjustment strategy focused on biologically relevant offspring characteristics while acknowledging the possibility of residual confounding.

### 2.3. Dietary Assessment

Maternal dietary intake during pregnancy was assessed using a semi-quantitative Food Frequency Questionnaire (FFQ) administered prospectively during routine prenatal visits. The FFQ evaluated habitual dietary intake over the preceding trimester and included the principal food groups characterizing Mediterranean dietary patterns. Dietary information was transformed into a Mediterranean Diet Score according to predefined scoring criteria, with higher scores indicating greater adherence to the Mediterranean dietary pattern. Dietary pattern analysis was selected because it better reflects the combined biological effects of foods and nutrients than analyses based on isolated nutritional components. Offspring adherence to the Mediterranean diet at follow-up was assessed using the KIDMED index, a validated instrument for evaluating adherence to Mediterranean dietary patterns in children and adolescents [22]. Higher KIDMED scores indicate greater adherence to the Mediterranean diet. The offspring KIDMED score was included in the sensitivity analyses to examine whether the observed associations between maternal Mediterranean diet adherence and autism-related traits were independent of the child’s contemporary dietary habits.

The Mediterranean Diet Score was analysed as a continuous variable throughout all statistical analyses. No arbitrary cut-off values were applied to classify participants into high- or low-adherence categories because this approach preserves statistical power and minimizes information loss.

### 2.4. Assessment of Autism-Related Traits

Autism-related traits were assessed during follow-up using the Australian Scale for Asperger’s Syndrome (ASAS), a parent-completed screening instrument developed by Attwood to identify behavioral and developmental characteristics associated with Asperger syndrome and autism spectrum traits in school-aged children [23].

The ASAS evaluates multiple domains of functioning, including social and emotional abilities, communication skills, cognitive characteristics, special interests, and motor skills. Parents rate the frequency and severity of specific behaviors, with higher scores indicating greater expression of autism-spectrum characteristics. The ASAS was used exclusively as a screening instrument for dimensional autism-related traits and was not intended to establish a clinical diagnosis of Autism Spectrum Disorder.

For the purposes of the present study, domain-specific scores were calculated for Social and Emotional Abilities, Communication Skills, Cognitive Characteristics, Special Interests, and Motor Skills. Higher scores reflected greater expression of autism-related traits within each domain. A composite autism-related trait score was additionally calculated as the sum of all domain scores and was used for descriptive purposes only. Primary analyses focused on individual trait domains, particularly communication-related traits, rather than on clinical classification or diagnosis.

### 2.5. Statistical Analysis

Continuous variables are presented as mean ± standard deviation (SD), whereas categorical variables are presented as frequencies and percentages. Differences between boys and girls were evaluated using independent-samples *t*-tests. Associations between the maternal Mediterranean Diet Score during pregnancy and autism-related trait domains were examined using Spearman’s rank correlation coefficients. Sex-stratified analyses were subsequently performed to investigate the potential differences between male and female offspring.

Given the exploratory nature of the present study and the relatively modest sample size, we deliberately adopted parsimonious multivariable models to reduce overfitting. Covariates were selected a priori based on biological plausibility and existing literature rather than automated statistical selection procedures. Model complexity was intentionally limited to preserve statistical stability.

Covariate selection was guided by biological plausibility and a prespecified causal framework. A Directed Acyclic Graph (DAG) was constructed to identify variables considered potential confounders while avoiding unnecessary adjustment for mediators or collider variables.

Prior to model estimation, assumptions of linear regression were evaluated. Linearity between predictors and outcome was assessed graphically. Residual normality was examined using Q-Q plots and the Shapiro–Wilk test. Homoscedasticity was evaluated through residual-versus-fitted plots. Multicollinearity was assessed using variance inflation factors, while influential observations were examined using Cook’s distance. No substantial violations of model assumptions were identified.

Missing values were handled using complete-case analysis. Participants with missing values for variables included in a given statistical model were excluded only from that specific analysis. Consequently, sample sizes differed slightly across analyses. Given the exploratory nature of the study and the relatively limited number of missing observations, multiple imputation was not performed. Before performing sex-stratified analyses, interaction between offspring sex and maternal Mediterranean Diet Score was evaluated by including a multiplicative interaction term in the regression model. Sample sizes presented in each model correspond to participants with complete information for all variables included in that model. Adjusted regression analyses presented in the Supplementary Materials were performed using complete-case analysis; therefore, sample sizes varied across models depending on the availability of complete data for all included covariates.

Sequential linear regression models were constructed among boys to further explore the association between maternal Mediterranean Diet Score and communication-related traits. Models were adjusted for child BMI, sleep duration, and physical activity level (PAQ score). Regression coefficients (β), 95% confidence intervals (CI), and coefficients of determination (R^2^) are reported. Statistical significance was set at a two-sided *p*-value < 0.05. All analyses were performed using the SPSS software 2021 (IBM Corp., Armonk, NY, USA).

## 3. Results

### 3.1. Participant Characteristics

A total of 96 children with available maternal dietary and offspring neurodevelopmental data were included in the present analysis (49 boys and 47 girls). Maternal adherence to the Mediterranean diet during pregnancy did not differ between boys and girls (30.15 ± 6.16 vs. 30.03 ± 4.37, *p* = 0.937). Similarly, no sex differences were observed in BMI or physical activity levels. In contrast, girls reported longer sleep duration than boys (9.52 ± 0.90 vs. 8.87 ± 0.82 h/day, *p* = 0.008) (Table 1).

**Figure 1:**
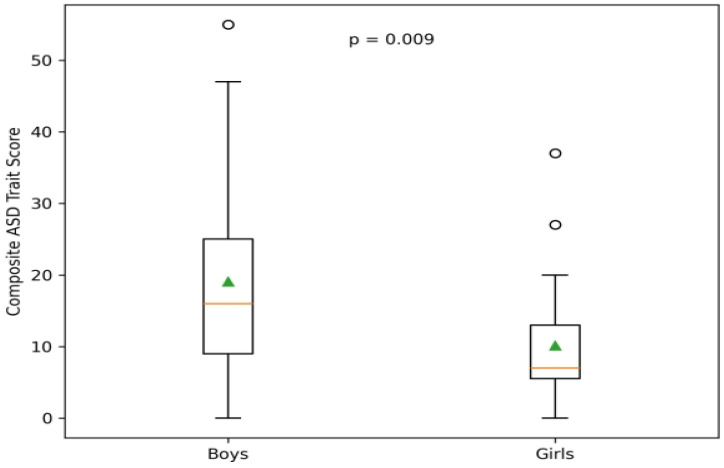
Sex differences in autism-related trait burden. Boys exhibited higher composite autism-related trait scores than girls (19.08 ± 13.92 vs. 10.15 ± 8.57; *p* = 0.009). Horizontal lines represent median, green triangles mean and open circles outlier values. ASD: autism spectrum disorder.

Regarding autism-related traits, boys exhibited higher scores than girls in several domains. Communication trait scores were more than two-fold higher in boys (3.92 ± 3.90 vs. 1.70 ± 2.89, *p* = 0.026). Similarly, boys demonstrated higher cognitive trait scores (5.76 ± 3.80 vs. 3.33 ± 2.99, *p* = 0.014) and higher special-interest scores (2.64 ± 3.29 vs. 1.04 ± 2.16, *p* = 0.046). Although social-emotional trait scores tended to be higher among boys, the difference did not reach statistical significance (6.24 ± 6.84 vs. 3.59 ± 5.32, *p* = 0.128). No sex differences were observed for motor-skill traits (*p* = 0.906). Overall, boys displayed a higher autism-related trait burden than girls, as reflected by the composite ASD-trait score (19.08 ± 13.92 vs. 10.15 ± 8.57, *p* = 0.009) (Figure 1).

### 3.2. Associations Between Maternal Mediterranean Diet Adherence and Autism-Related Traits

Spearman correlation analyses between the maternal Mediterranean Diet Score during pregnancy and autism-related traits are presented in Table 2. In the overall cohort, maternal Mediterranean diet adherence was not associated with any autism-related trait domain. Correlation coefficients were generally weak and non-significant, suggesting no overall association when boys and girls were analyzed together.

**Figure 2:**
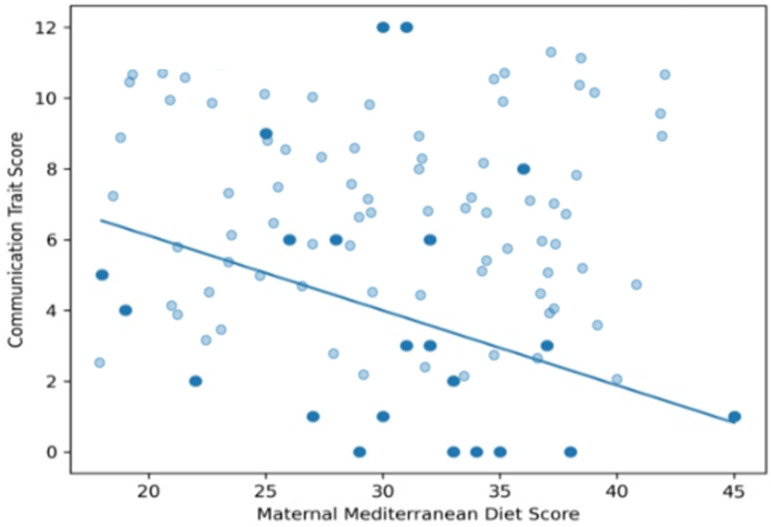
Association between maternal Mediterranean Diet Score during pregnancy and communication-related autism traits among male offspring. Higher maternal Mediterranean Diet Scores were associated with lower communication-trait scores (Spearman ρ = −0.442, *p* = 0.027).

In sex-stratified analyses, an inverse association was observed between maternal Mediterranean Diet Score and communication-related traits among boys (ρ = −0.442, *p* = 0.027) (Figure 2). No associations were observed for social-emotional traits (ρ = −0.386, *p* = 0.056), cognitive traits (ρ = −0.349, *p* = 0.087), special interests (ρ = −0.337, *p* = 0.099), or motor-skill traits (ρ = −0.024, *p* = 0.908). Among girls, maternal Mediterranean Diet Score was not associated with communication, cognitive, special-interest, or motor-skill traits. Overall, communication-related traits represented the only domain demonstrating an association with maternal Mediterranean diet adherence among boys.

Regression analyses among girls demonstrated no statistically significant associations between maternal Mediterranean Diet Score and communication-related traits. Similarly, analyses performed in the overall cohort yielded null findings. These analyses are presented in Supplementary Tables S1–S4.

No statistically significant association was observed in the overall cohort. However, a sex-by-Mediterranean Diet Score interaction suggested heterogeneity of effect according to offspring sex, supporting subsequent stratified analyses.

### 3.3. Multivariable Regression Analyses

To further explore the association between maternal Mediterranean diet adherence and communication-related traits, sequential linear regression models were performed among boys (Table 3). Across all models, higher maternal Mediterranean Diet Scores were consistently associated with lower communication-trait scores. Although the magnitude of the association remained relatively stable after adjustment for BMI, sleep duration, and physical activity, statistical significance was not achieved in either the crude or adjusted models.

In crude analyses, higher maternal Mediterranean Diet Scores were associated with lower communication-trait scores (β = −0.211, 95% CI −0.473 to 0.050, *p* = 0.108). Adjustment for BMI produced similar estimates (β = −0.234, *p* = 0.140), while additional adjustment for sleep duration resulted in minimal change in the magnitude of the association (β = −0.255, *p* = 0.106). Following further adjustment for physical activity, the inverse association remained evident, although it did not reach statistical significance (β = −0.293, 95% CI −0.713 to 0.127, *p* = 0.157). Overall, regression analyses demonstrated a consistent inverse direction of association between maternal Mediterranean diet adherence and communication-related autism traits among boys, although the association was attenuated after multivariable adjustment.

### 3.4. Sensitivity Analysis Including Offspring KIDMED Score

Additional sensitivity analyses were performed by including offspring KIDMED scores in the regression models. KIDMED scores did not differ between boys and girls (5.48 ± 2.87 vs. 6.45 ± 2.18, *p* = 0.165), and maternal Mediterranean Diet Score was not correlated with offspring KIDMED score. Importantly, the inclusion of offspring KIDMED scores in multivariable models did not materially alter the inverse association between maternal Mediterranean Diet Score and communication-related traits among boys. These findings suggest that the observed association was not explained by the offspring’s contemporary adherence to the Mediterranean diet.

## 4. Discussion

The present exploratory analysis of the KLOTHO birth cohort investigated whether maternal adherence to the Mediterranean diet during pregnancy is associated with autism-related traits in offspring during preadolescence.

The principal findings were threefold. First, male offspring exhibited higher autism-related trait scores than female offspring across several domains, including communication, cognitive characteristics, and special interests. Second, no associations were observed between adherence to the Mediterranean diet and autism-related traits when the entire cohort was analyzed as a single group. Third, sex-stratified analyses revealed that higher maternal adherence to the Mediterranean diet during pregnancy was associated with lower communication-related trait scores in boys than in girls.

The observation that boys demonstrated higher autism-related trait scores than girls is consistent with a substantial body of literature indicating male predominance in autism spectrum characteristics. Epidemiological studies have consistently reported higher prevalence rates of autism spectrum disorder among males, with male-to-female ratios commonly ranging from 3:1 to 4:1 [1,2,3,4]. Beyond clinical diagnoses, autism-related characteristics are more frequently observed among boys in community-based samples.

Several mechanisms have been proposed to explain sex differences in autism. The “female protective effect” hypothesis suggests that females may require a greater genetic or environmental burden before manifesting autism-related characteristics [2,3]. Furthermore, sex-specific neurodevelopmental trajectories, hormonal influences, and differences in fetal brain maturation may contribute to the increased vulnerability observed in males. Consequently, the higher communication, cognitive, and special-interest scores observed among boys in the present cohort are broadly consistent with the current understanding of autism-related trait distribution within the general population.

The most notable finding of the present study was the inverse association between adherence to the Mediterranean diet during pregnancy and communication-related autism traits among male offspring [19]. Communication difficulties are one of the core dimensions of autism-related behavioral characteristics. Therefore, the observed association may indicate that prenatal nutritional exposure influences the developmental pathways related to social communication and language-related functioning.

Although the study design does not permit causal inference, several biological mechanisms could explain the observed association. The Mediterranean diet is characterized by a high consumption of fruits, vegetables, legumes, whole grains, olive oil, nuts, and fish, resulting in an increased intake of nutrients known to support fetal neurodevelopment [12,13,14,15].

Among these nutrients, folate plays a critical role in DNA synthesis, methylation reactions, and neural tube development. Adequate maternal folate status has repeatedly been associated with improved neurodevelopmental outcomes in offspring [16]. Similarly, omega-3 polyunsaturated fatty acids, particularly DHA, contribute to neuronal membrane integrity, synaptogenesis, and cognitive development [17].

In addition, the Mediterranean dietary pattern provides substantial quantities of antioxidants and (poly)phenols. These bioactive components may reduce oxidative stress and inflammatory signaling during pregnancy, both of which have been implicated in altered neurodevelopmental trajectories in offspring. Experimental evidence suggests that prenatal inflammation may influence fetal brain development, particularly in pathways associated with social behavior and communication [18]. Taken together, these mechanisms provide biological plausibility for the inverse association observed between adherence to the Mediterranean diet during pregnancy and communication-related autism traits among boys.

An important aspect of these findings is that associations were primarily observed among male offspring. Previous research suggests that male fetuses may be more susceptible to environmental exposure during critical developmental periods. Prenatal nutritional factors, inflammatory stimuli, and metabolic disturbances have all been reported to exert stronger neurodevelopmental effects in males than in females [2,3,20].

One possible explanation is that the developing male brain demonstrates greater sensitivity to both adverse and beneficial prenatal exposures. Consequently, positive nutritional influences during pregnancy may be more readily detectable in boys, particularly in domains related to communication and social functioning [2,3]. However, the present findings should be interpreted cautiously. Although sex-stratified analyses suggested stronger associations among boys, the relatively small sample size limits statistical power and increases uncertainty around effect estimates. Therefore, these observations should be regarded as hypothesis-generating rather than definitive evidence of sex-specific biological mechanisms.

Research examining maternal dietary patterns and autism-related outcomes remains relatively limited. Most previous investigations have focused on specific nutrients, such as folate, vitamin D, omega-3 fatty acids, as well as overall dietary quality during pregnancy [16,17,18,24,25,26,27]. Several cohort studies have reported favorable associations between adherence to healthy dietary patterns and offspring cognitive or behavioral outcomes [5,6,7,8,9]. However, relatively few studies have specifically examined autism-related traits during later childhood or preadolescence [19]. The present findings contribute to this emerging literature by extending the investigation to autism-related communication traits and by evaluating outcomes approximately 12 years after the prenatal exposure period. Although the observed associations require replication, they are broadly consistent with the hypothesis that prenatal dietary quality may influence long-term neurodevelopmental trajectories. Socioeconomic position is an important determinant of maternal diet quality and childhood neurodevelopment. Although maternal educational level was available for part of the cohort, incomplete data precluded its inclusion in the final adjusted models. Larger prospective cohorts with comprehensive socioeconomic characterization will be necessary to determine whether the observed associations remain independent of maternal educational attainment and other social determinants of health. Although maternal educational level was considered during model development, incomplete data availability precluded its inclusion in the final multivariable models. Therefore, some degree of residual socioeconomic confounding cannot be excluded.

Our findings should also be interpreted within the broader literature investigating maternal dietary patterns and offspring neurodevelopment. Several prospective cohort studies have reported that greater adherence to healthy dietary patterns during pregnancy is associated with improved cognitive performance, executive function, behavioural regulation and emotional development in childhood. Nevertheless, evidence specifically addressing autism-related traits remains considerably more limited and inconsistent. Therefore, the present findings extend existing knowledge by suggesting that maternal Mediterranean dietary adherence may be associated with specific dimensions of neurodevelopment, particularly communication-related traits among male offspring, although these observations require confirmation in larger independent cohorts.

The observed sex-specific association is biologically plausible given increasing evidence that male fetal neurodevelopment may be more susceptible to prenatal nutritional and inflammatory exposures. Nevertheless, because our analyses were exploratory and based on a relatively modest sample size, these findings should be interpreted cautiously until replicated in adequately powered prospective studies. Because this represents a secondary analysis of an existing birth cohort, no prospective sample-size calculation was performed for the present research question. Residual socioeconomic confounding remains possible because maternal educational attainment and other socioeconomic indicators could not be fully incorporated into the adjusted analyses. Consequently, the observed associations should be interpreted cautiously until replicated in larger cohorts with more comprehensive socioeconomic characterization. Nevertheless, adjustment for an excessive number of covariates in a relatively small sample may introduce model instability and overfitting.

The present findings were obtained in a Mediterranean population from Northern Greece and therefore should not automatically be generalized to populations with substantially different dietary patterns, cultural practices or socioeconomic characteristics. Future multicentre prospective cohorts including geographically diverse populations will be important to determine the external validity and reproducibility of these observations.

The observed sex-specific association should not be interpreted as evidence of a causal biological mechanism. Because our analyses were based on an overall dietary pattern rather than individual nutrient intake, mechanistic inferences cannot be made directly from the present data. Nevertheless, increasing experimental and epidemiological evidence suggests that male fetal neurodevelopment may be more susceptible to prenatal nutritional and inflammatory exposures through sex-specific placental function, immune regulation and epigenetic programming. These mechanisms provide biological plausibility for our observations but require confirmation in dedicated mechanistic studies.

The present study had several strengths. First, the prospective birth cohort design allowed for the assessment of maternal dietary exposure during pregnancy prior to the measurement of offspring outcomes, thereby reducing the possibility of reverse causation. Second, autism-related traits were assessed during preadolescence, providing insight into longer-term developmental outcomes rather than just early childhood behaviors. Third, this study examined multiple autism-related domains, including communication, social-emotional abilities, cognitive characteristics, special interests, and motor skills.

An additional strength of the study is that the observed associations were examined after considering offspring adherence to the Mediterranean diet at follow-up. The inverse association between maternal Mediterranean diet adherence and communication-related traits among boys remained largely unchanged, suggesting that the findings were not simply attributable to the contemporary dietary habits of offspring. Importantly, adjustment for offspring adherence to the Mediterranean diet did not materially alter the observed associations, supporting the hypothesis that prenatal rather than contemporary dietary exposures may contribute to the observed findings.

Nevertheless, several limitations of this study should be acknowledged. The most important limitation of this study is the relatively small sample size, particularly for sex-stratified analyses. The limited statistical power increases the possibility of both false-positive and false-negative findings. Second, autism-related traits were evaluated using questionnaire-based assessments rather than clinical diagnoses. Consequently, this study examined dimensional autism-related characteristics rather than the autism spectrum disorder itself. Third, residual confounding cannot be excluded, despite adjustments for the selected child characteristics. Additional factors, including socioeconomic status, parental education, maternal health, and other environmental influences, may contribute to the observed associations. Although several maternal characteristics were collected during pregnancy, maternal educational level was not included in the final multivariable models because of incomplete data availability and concerns regarding overfitting given the modest sample size. Consequently, residual confounding related to socioeconomic status cannot be excluded. Sensitivity analyses were limited to adjustment for offspring adherence to the Mediterranean diet because alternative autism assessment instruments and repeated neurodevelopmental evaluations were not available within the cohort. Future studies incorporating multiple standardized neurodevelopmental instruments may provide more comprehensive validation of these findings.

Because autism-related traits were evaluated using a parent-completed screening questionnaire rather than standardized diagnostic assessments, the present findings should not be interpreted as evidence regarding clinically diagnosed Autism Spectrum Disorder. Instead, they reflect variation in autism-related characteristics within the general population.

The complete-case approach substantially reduced the sample size in the adjusted sensitivity analyses, particularly in subgroup analyses, which may have limited statistical power and the precision of effect estimates. Therefore, these findings should be interpreted as exploratory and supportive of the primary analyses rather than conclusive.

Finally, the exploratory nature of the analyses, the relatively small sample size, and the multiple correlation tests performed increase the possibility of both false-positive and false-negative findings. Therefore, the observed associations should be considered hypothesis-generating and require independent replication in larger prospective cohorts. The relatively modest sample size, especially within the sex-stratified analyses, limited statistical power and increased uncertainty around the effect estimates.

## 5. Conclusions

In this exploratory analysis of the KLOTHO birth cohort, higher maternal adherence to the Mediterranean diet during pregnancy was associated with lower communication-related trait scores among male offspring. Boys exhibited higher autism-related trait scores than girls across several domains, consistent with previous literature. Although no associations were observed in the overall cohort, the sex-specific findings suggest that prenatal dietary quality may influence communication-related developmental outcomes in males. Given the modest sample size and exploratory design, these results should be interpreted as hypothesis-generating only and should not be interpreted as evidence that maternal Mediterranean diet adherence prevents or modifies clinically diagnosed Autism Spectrum Disorder.

Future investigations should include multicenter prospective birth cohorts with larger sample sizes, repeated neurodevelopmental assessments across childhood, comprehensive characterization of maternal socioeconomic and environmental factors, standardized clinical diagnostic instruments for Autism Spectrum Disorder, and integration of nutritional biomarkers, inflammatory mediators and epigenetic profiling to clarify the biological mechanisms underlying the observed associations.

## Figures and Tables

**Table 1:** Participant characteristics according to sex.

	Boys		Girls		
Variable	*n*	Mean ± SD	*n*	Mean ± SD	*p*-Value
Maternal MedDiet Score	49	30.15 ± 6.16	47	30.03 ± 4.37	0.937
Child BMI (kg/m^2^)	44	17.81 ± 3.68	46	17.39 ± 3.71	0.696
Sleep duration (h/day)	47	8.87 ± 0.82	47	9.52 ± 0.90	0.008
PAQ score	49	2.80 ± 0.57	46	2.91 ± 0.66	0.519
Social and Emotional Traits	48	6.24 ± 6.84	45	3.59 ± 5.32	0.128
Communication Traits	47	3.92 ± 3.90	47	1.70 ± 2.89	0.026
Cognitive Traits	49	5.76 ± 3.80	47	3.33 ± 2.99	0.014
Special Interests	49	2.64 ± 3.29	46	1.04 ± 2.16	0.046
Motor Skills	49	0.52 ± 1.12	47	0.48 ± 1.22	0.906
Composite ASD Trait Score	49	19.08 ± 13.92	47	10.15 ± 8.57	0.009

ASD: autism spectrum disorder; BMI: body mass index; PAQ: Physical Activity Questionnaire; SD: standard deviation. Sample sizes differ across variables because complete-case analysis was used for each individual analysis.

**Table 2:** Spearman correlations between maternal Mediterranean Diet score and autism-related traits.

	Overall			Boys			Girls		
Outcome	*n*	ρ	*p*	*n*	ρ	*p*	*n*	ρ	*p*
Social and Emotional Traits	96	−0.026	0.855	49	−0.386	0.056	47	0.360	0.065
Communication Traits	96	−0.168	0.233	49	−0.442	0.027	47	0.169	0.399
Cognitive Traits	96	−0.124	0.380	49	−0.349	0.087	47	0.146	0.468
Special Interests	96	−0.034	0.810	49	−0.337	0.099	46	0.344	0.079
Motor Skills	96	−0.019	0.896	49	−0.024	0.908	47	−0.020	0.920
Composite ASD Trait Score	96	−0.103	0.469	49	−0.528	0.007	47	0.417	0.030

ASD: autism spectrum disorder; ρ = Spearman’s rank correlation coefficient. Negative coefficients indicate lower trait scores with higher maternal MedDiet score.

**Table 3:** Sequential linear regression models for communication traits in boys.

Model	*n*	β	95% CI	*p*-Value	R^2^
Crude	47	−0.211	−0.473 to 0.050	0.108	0.108
+ BMI	47	−0.234	−0.552 to 0.084	0.140	0.122
+ BMI + sleep	46	−0.255	−0.569 to 0.060	0.106	0.207
+ BMI + sleep + Physical Activity Score	44	−0.293	−0.713 to 0.127	0.157	0.226

BMI: body mass index; CI: confidence interval; β values are unstandardized coefficients per 1-point increase in the maternal MedDiet score.

## Data Availability

The data presented in this study are available upon reasonable request from the corresponding author.

## Supplementary Materials

The following supporting information can be downloaded at: https://www.mdpi.com/article/10.3390/nu18142256/s1, Figure S1: Directed Acyclic Graph (DAG); Table S1: Overall cohort: adjusted multivariable linear regression; Table S2: Boys: adjusted multivariable linear regression; Table S3: Girls: adjusted multivariable linear regression; Table S4: Formal sex interaction model.
