# Supplementary material for: Maternal Mediterranean Diet Adherence During Pregnancy and Autism-Related Traits in Preadolescence: A Sex-Stratified Analysis
Source: Nutrients. 2026 Jul 10;18(14):2256. doi: 10.3390/nu18142256 (PMC13415154; doi:10.3390/nu18142256)
Supplement: Supplementary file 1 [file nutrients-18-02256-s001.zip › nutrients-4392398-supplementary.pdf]

Additional Multivariable Regression Analyses

Outcome: communication-related autism traits (ASAS communication domain).

Data handling and model specification

- Analyses were performed using the individual-level data from the uploaded KLOTHO database, not aggregate values from the manuscript tables.
- Child BMI was calculated from the child height and weight variables in the database: weight (kg) / height $(m)^2$.
- Sex was coded as male sex = 1 for boys and 0 for girls, based on the dataset coding where Sex = 1 corresponds to boys and Sex = 2 corresponds to girls.
- Sleep duration was taken from the hours of sleep variable. Entries stored by Excel as dates were recoded using the day component because the intended values were sleep hours.
- Models used complete-case analysis; therefore, sample size differs across models according to available data for the included variables.
- For the interaction model, maternal MedDiet Score was mean-centered within the complete interaction-model sample (mean = 30.465) before constructing the interaction term. This preserves the interaction estimate while reducing non-essential collinearity.

Model fit summary

Model	n	df model	df residual	R^2	Adjusted R^2	F statistic	Model p-value
Overall cohort	43	5	37	0.307	0.213	3.275	0.015
Boys	20	4	15	0.285	0.095	1.496	0.253
Girls	23	4	18	0.521	0.415	4.894	0.008
Interaction	43	6	36	0.368	0.263	3.501	0.008

Supplementary Table S1. Overall cohort: adjusted multivariable linear regression

Model characteristics: n = 43; R^2 = 0.307; adjusted R^2 = 0.213; F(5, 37) = 3.275; model p-value = 0.015.

Predictor	B	SE	Std. β	95% CI	p-value	VIF
Maternal MedDiet Score	-0.164	0.104	-0.220	-0.375 to 0.046	0.123	1.034
Male sex	3.970	1.125	0.540	1.691 to 6.248	0.001	1.248
Child BMI	0.177	0.147	0.179	-0.121 to 0.474	0.236	1.180
Sleep duration	1.317	0.548	0.363	0.207 to 2.427	0.021	1.215
Physical activity score (PAQ)	0.268	0.867	0.046	-1.489 to 2.026	0.759	1.187

B values are unstandardized regression coefficients. Std. β values are standardized coefficients. VIF = variance inflation factor.

Supplementary Table S2. Boys: adjusted multivariable linear regression

Model characteristics: n = 20; R^2 = 0.285; adjusted R^2 = 0.095; F(4, 15) = 1.496; model p-value = 0.253.

Predictor	B	SE	Std. β	95% CI	p-value	VIF
Maternal MedDiet Score	-0.322	0.188	-0.422	-0.723 to 0.078	0.107	1.268

Child BMI	0.002	0.262	0.002	-0.556 to 0.560	0.994	1.192
Sleep duration	1.947	1.210	0.381	-0.631 to 4.525	0.128	1.176
Physical activity score (PAQ)	-0.054	1.888	-0.007	-4.078 to 3.970	0.978	1.413

B values are unstandardized regression coefficients. Std. β values are standardized coefficients. VIF = variance inflation factor.

Supplementary Table S3. Girls: adjusted multivariable linear regression

Model characteristics: n = 23; R^2 = 0.521; adjusted R^2 = 0.415; F(4, 18) = 4.894; model p-value = 0.008.

Predictor	B	SE	Std. β	95% CI	p-value	VIF
Maternal MedDiet Score	0.129	0.112	0.208	-0.105 to 0.364	0.262	1.214
Child BMI	0.507	0.140	0.692	0.213 to 0.801	0.002	1.368
Sleep duration	0.678	0.450	0.253	-0.268 to 1.623	0.149	1.063
Physical activity score (PAQ)	1.994	0.803	0.500	0.306 to 3.681	0.023	1.527

B values are unstandardized regression coefficients. Std. β values are standardized coefficients. VIF = variance inflation factor.

Supplementary Table S4. Formal sex interaction model

Model characteristics: n = 43; R^2 = 0.368; adjusted R^2 = 0.263; F(6, 36) = 3.501; model p-value = 0.008.

Predictor	B	SE	Std. β	95% CI	p-value	VIF
Maternal MedDiet Score (centered)	0.073	0.162	0.098	-0.255 to 0.401	0.653	2.672
Male sex	4.017	1.088	0.546	1.809 to 6.224	<0.001	1.249
Maternal MedDiet Score × Male sex	-0.419	0.224	-0.420	-0.873 to 0.034	0.069	2.855
Child BMI	0.204	0.143	0.207	-0.085 to 0.494	0.161	1.192
Sleep duration	1.314	0.530	0.362	0.239 to 2.389	0.018	1.215
Physical activity score (PAQ)	0.999	0.925	0.172	-0.878 to 2.875	0.288	1.443

B values are unstandardized regression coefficients. Std. β values are standardized coefficients. VIF = variance inflation factor.

Following the Reviewer's recommendation, additional multivariable regression analyses were performed in the overall cohort and separately among boys and girls. In the overall cohort, maternal Mediterranean Diet Score was not significantly associated with communication-related autism traits after adjustment for child BMI, sleep duration and physical activity (B = -0.164, 95% CI -0.375 to 0.046, p-value = 0.123), whereas male sex was independently associated with higher communication-trait scores (B = 3.970, 95% CI 1.691 to 6.248, p-value = 0.001) (Supplementary Table S1). In sex-stratified analyses, the association between maternal Mediterranean Diet Score and communication-related traits was inverse among boys, although it did not reach statistical significance after multivariable adjustment (B = -0.322, 95% CI -0.723 to 0.078, p-value = 0.107) (Supplementary Table S2). No significant association was observed among girls (B = 0.129, 95% CI -0.105 to 0.364, p-value = 0.262) (Supplementary Table S3). A formal interaction model suggested a trend toward sex-specific effect modification for the association between maternal Mediterranean Diet Score and communication-related traits (interaction B = -0.419, 95% CI -0.873 to 0.034, p-value = 0.069), although this interaction did not reach conventional statistical significance (Supplementary Table S4).

Supplementary Figure S1. Directed Acyclic Graph (DAG)

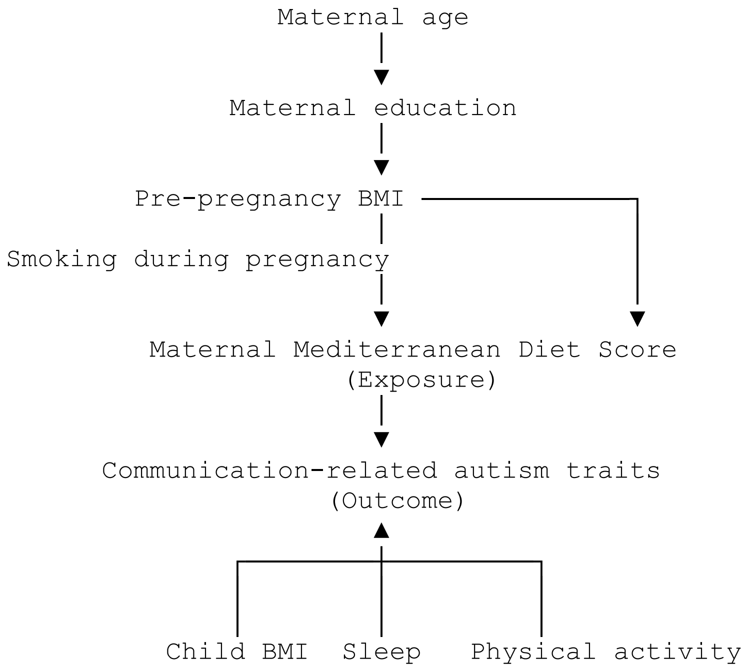

Figure S1. Directed acyclic graph illustrating the hypothesized causal framework underlying the association between maternal Mediterranean Diet Score during pregnancy (exposure) and offspring communication-related autism traits (outcome). Maternal age, maternal education, smoking during pregnancy and pre-pregnancy BMI were considered potential confounders. Child BMI, sleep duration and physical activity were included as covariates in the adjusted regression analyses.
